# Synthesizing evidence to guide the design and implementation of effective strategies for discontinuing postoperative antibiotic prophylaxis in surgical settings: an umbrella review post-WHO 2018 recommendations

**DOI:** 10.1186/s13643-024-02750-7

**Published:** 2025-01-08

**Authors:** George Msema Bwire, Renatus B. Magati, Hafidhi H. Ntissi, Tusaligwe Mbilinyi, Martine A. Manguzu, Goodluck G. Nyondo, Belinda J. Njiro, Lilian B. Nkinda, Castory G. Munishi, Obadia Nyongole, Pacifique Ndayishimiye, Mtebe V. Majigo

**Affiliations:** 1 Department of Pharmaceutical Microbiology, Muhimbili University of Health and Allied Science, Dar Es Salaam, Tanzania; 2 Department of Clinical Nursing, Catholic University of Health and Allied Sciences, Mwanza, Tanzania; 3 Department of Clinical Pharmacy and Pharmacology, Muhimbili University of Health and Allied Science, Dar Es Salaam, Tanzania; 4 Department of Medicinal Chemistry, Muhimbili University of Health and Allied Science, Dar Es Salaam, Tanzania; 5 Department of Epidemiology and Biostatistics, Muhimbili University of Health and Allied Science, Dar Es Salaam, Tanzania; 6 Department of Microbiology and Immunology, Muhimbili University of Health and Allied Science, Dar Es Salaam, Tanzania; 7 Department of Pharmaceutics and Pharmacy Practice, Muhimbili University of Health and Allied Science, Dar Es Salaam, Tanzania; 8 Department of Surgery, Muhimbili University of Health and Allied Science, Dar Es Salaam, Tanzania; 9 Department of Pharmacy, College of Medicine and Health Sciences, University of Rwanda, P.O. Box 4285, Kigali, Rwanda

**Keywords:** Postoperative antibiotic prophylaxis, Surgical site infection, Best practice, Strategies

## Abstract

**Background:**

Postoperative antibiotic prophylaxis (PAP) involves using antibiotics after surgery to prevent surgical site infections (SSIs). However, studies have shown that PAP offers no additional benefits compared to discontinuation after surgical incision closure, prompting its de-implementation to prevent unnecessary antibiotic use that may contribute to antibiotic resistance. We conducted this review to synthesize evidence for guiding the design and implementation of effective strategies for discontinuing PAP practice and optimizing antibiotic use in surgical settings.

**Methods:**

This umbrella review searched for articles from PubMed/MEDLINE and Scopus, focusing on reviews conducted on human subjects on PAP to prevent SSIs, published in English language from 2019 to 5th July 2024. This review followed guidelines from PRISMA-P and PRIOR. The risk of bias (methodological quality) was assessed using AMSTAR-2. The pooled risk ratio (RR) was estimated using a fixed-effects model (Mantel–Haenszel method), while *I*^2^ was used to assess the heterogeneity between reviews. This review was registered with PROSPERO (CRD42024566124).

**Results:**

In our umbrella review, we screened 1156 articles, with 28 review articles found eligible for final analysis, involving over 457 primary studies. About 80,483 patients were involved in 9 meta-analysis reviews, which were used to estimate the pooled RR. We found no significant benefits to patients from continuing PAP beyond 24-h post-surgery compared to immediate discontinuation, *RR*: 1.07 (95% *CI*: 0.97–1.17, *I*^2^: 25%, *p*-value: 0.22). Strategies such as regularly assessing and refining guidelines to fit specific surgical settings and patients’ characteristics, multidisciplinary collaboration, availability of resources needed for best practices, education and training healthcare workers on SSI prevention and antibiotic stewardship, and patient education in SSI prevention and proper antibiotic use were recommended to improve best practices in surgical settings.

**Conclusions:**

Prolonging antibiotic prophylaxis beyond 24-h post-surgery did not show significant protective benefits against SSIs. Our findings support the 2018 WHO recommendation for the immediate discontinuation of PAP following surgical incision closure in clean and clean-contaminated procedures. Further de-implementation research studies are needed to guide the effective discontinuation of PAP practice.

**Supplementary Information:**

The online version contains supplementary material available at 10.1186/s13643-024-02750-7.

## Background

Surgical site infection (SSI) is an infection occurring after surgery in the part of the body where the procedure was performed [1]. SSI rates can vary widely, affecting approximately 2 to 20% of surgical patients. The highest incidence rate is observed in hepatobiliary surgeries at 19% (95% *CI*: 15–23%), while the lowest rate is seen in gallbladder surgeries at 3% (95% *CI*: 2–4%) [2]. Given the millions of surgical procedures performed annually worldwide [3], the financial and social burden of SSIs is substantial, leading to prolonged hospital stays, additional surgical procedures, and increased healthcare costs [4]. Evidence-based interventions, such as antibiotic prophylaxis, are necessary to reduce the risk of SSIs and mitigate their impact on patients and healthcare systems [5]. Antibiotic prophylaxis can be administered in three stages: preoperatively, intraoperatively, and postoperatively [6].

Preoperatively, antibiotics are administered to achieve optimal tissue concentrations by the time of incision, effectively targeting potential pathogens and thereby minimizing the risk of SSIs [7]. Intraoperatively, antibiotics are maintained to sustain therapeutic levels throughout the procedure, bolstering infection control measures [8]. Postoperatively, antibiotics are administered to further prophylactically reduce the incidence of SSIs following surgery [6]. However, evidence suggests that continuing postoperative antibiotic prophylaxis (PAP) after surgical incision closure confers no additional benefits [9], potentially resulting in unnecessary antibiotic use [10]. This overuse can have profound consequences, including exacerbating antibiotic resistance, increased toxicity, bacterial superinfection, and imposing a financial burden on the patient and the health care system [11].

In response to the existing evidence, in 2018, the World Health Organization (WHO) [12] and other health organizations, such as the Centers for Disease Control and Prevention (CDC) [13], recommended best practices to ensure the appropriate use of antibiotic prophylaxis preoperatively, intraoperatively, and postoperatively. This includes the recommendation to discontinue antibiotic prophylaxis immediately after surgical incision closure, irrespective of the surgical site, for both clean and clean-contaminated surgeries [12, 13]. Despite guidelines recommending the cessation of PAP [12], the practice continues to persist [14–16]. Possible reasons for the continuation of this practice include surgeons’ fear of SSIs, leading them to opt for extended antibiotic use as a precautionary measure, local context, and strongly held traditional practice [17, 18]. Moreover, awareness gaps among healthcare providers regarding updated guidelines, coupled with patient expectations for antibiotics [19, 20], and inadequate knowledge about antibiotic stewardship contribute to PAP practice [21].

Furthermore, some of the evidence suggests that the decision to extend antibiotic prophylaxis beyond the intraoperative period should consider patient-specific factors [22] (e.g., immunocompromised individuals such as those undergoing organ transplant surgery or receiving chemotherapy) and the specific surgical site (e.g., joint replacements or abdominal procedures involving the gastrointestinal tract). In the context of the ongoing discussion about PAP [22–24], discontinuation of PAP practices can be viewed as a de-implementation challenge [25]. This necessitates regular updates informed by emerging evidence [24] and the development of effective and implementable interventions [26]. These strategies need to be tailored to the specific surgical settings and patient characteristics to ensure their adoption and effectiveness in preventing SSIs, thereby reducing reliance on PAP [27].

Given the current findings on PAP practice [22–24], there is a growing need for an umbrella review to comprehensively synthesize existing evidence [28], consolidating and evaluating the collective findings, especially those that have emerged after the WHO guidelines of 2018 [12]. Such an approach would help clarify the effectiveness of PAP across different patient populations and surgical scenarios [29–32] and explore strategies [33], guiding more informed clinical practices, policies, or interventions. Therefore, we designed this review to synthesize the current evidence following the WHO recommendations of 2018 on PAP practice.

## Methods

### Study design and protocol registration

This was an umbrella review (systematic review of reviews) [34] aimed at synthesizing evidence that can inform strategies for discontinuing PAP. Additionally, we pooled the effects from the meta-analysis reviews to compare the risk of acquiring SSIs between patients who continued PAP and those who discontinued it. The review protocol was prepared according to Preferred Reporting Items for Systematic review and Meta-Analysis Protocols (PRISMA-P) [35] and Preferred Reporting Items for Overviews of Reviews (PRIOR) [36]. This umbrella review was registered in the International Prospective Register of Systematic Reviews (PROSPERO: CRD42024566124).

### Search strategy

Articles were searched from PubMed/MEDLINE and Scopus [37]. We included reviews conducted in human subjects on PAP and published in English language from inception to 05th July 2024. The search strategy was initially developed for PubMed following guidance from Peer Review of Electronic Search Strategies (PRESS) [38] and subsequently adapted for Scopus. MeSH (Medical Subject Headings) terms were used to construct the PubMed search query, combining keywords with the Boolean operator “AND” to ensure inclusion of all specified criteria, while “OR” was used to retrieve results containing at least one of the keywords (Supplementary file 1). The search included keywords such as “antibiotic prophylaxis,” “surgical infection,” and “best practices.”

### Eligibility criteria

- Patient: Reviews involving human subjects who had undergone surgery and were considered for antibiotic prophylaxis to prevent SSI
- Intervention: Immediate discontinuation of PAP following surgical incision closure. Discontinuation of PAP was also assigned to patients who received antibiotics up to 24 h or less post-surgery.
- Comparator/control: The focus was on reviews that reported the continued use of PAP. Continuation of PAP was also assigned to patients who received preoperative antibiotics and continued on antibiotics for more than 24-h post-surgery.
- Outcome: Incidence of SSI [1] especially for meta-analysis or strategy to prevent SSI or appropriate use of antibiotics in surgical settings for narrative synthesis
- Study design: Systematic reviews, meta-analyses, scoping review, and literature reviews that reported the effect of both continuation and discontinuation of PAP in patients who underwent surgical procedures. Given that the WHO’s 2018 guideline on the prevention of SSI is based on systematic reviews of the evidence, this umbrella focused on reviews published after 2018 [12]. We also included reviews that reported strategies for best practices in preventing SSIs and the appropriate use of antibiotics in surgical settings [33].

### Study screening and data extraction

**Fig. 1:**
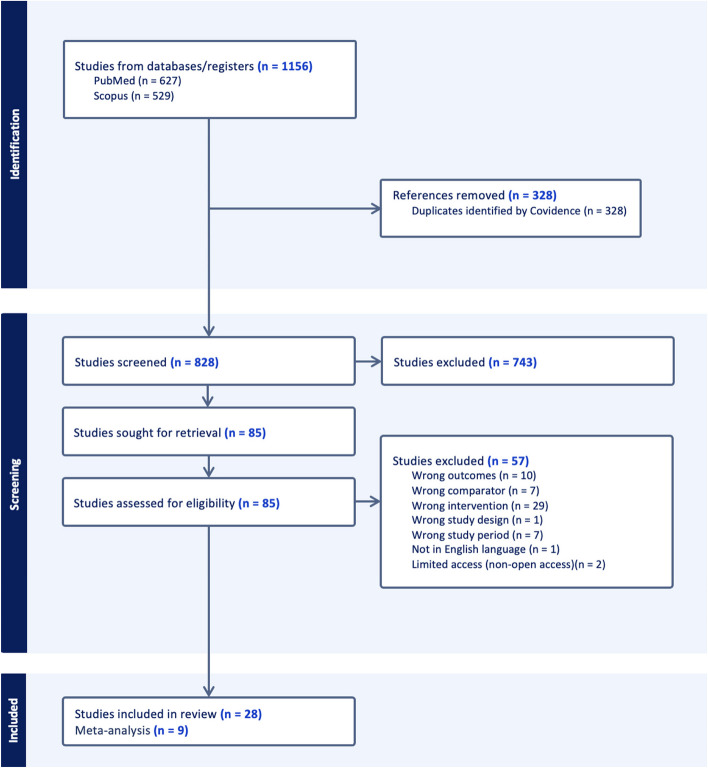
PRISMA flow diagram illustrating the study screening process, adopted from Covidence 2024

Two reviewers (R. B. M. and G. M. B.) independently conducted the screening based on the eligibility criteria and performed data extraction. Covidence software (Veritas Health Innovation, Melbourne, Australia) was used to manage the duplicates and perform title and abstract screening. Disagreements on screening and extraction were resolved by consulting the third author (M. V. M.). The selection procedures are outlined in Fig. 1. Full article screening was performed to extract information such as the first author’s surname and year of publication, type of review, and key findings (Table 1). We also extracted data on strategies or interventions or recommendations to prevent SSIs as outlined in Table 2. Furthermore, we extracted data on the incidence of SSIs for both discontinued and continued PAP to evaluate its effectiveness in preventing SSIs.

**Table 1:** Characteristics of the studies included in the umbrella review and summary of the key findings

Study name	Type of review	Surgical site	Key findings	^a^Methodological quality
Aldarragi 2023 [45]	SR	Breast	The incidence of SSIs does not significantly differ between the use of antibiotics for more than 24 h compared to their use for less than 24 h	Critically low
Białek (2023) [32]	SR	Urethra	Postoperative prophylaxis does not effectively prevent infections after hypospadias repair or urethroplasty in adults	Critically low
Blatt (2019) [46]	SR	Maxillofacial	For maxillofacial surgery, antibiotic prophylaxis may reduce SSIs, but extended postoperative dosing shows no benefit. In clean-contaminated oncological surgeries, a 24-h antibiotic regimen can effectively reduce SSIs without additional benefit from prolonged use	Critically low
Cooper (2020) [17]	Scoping review and MA	Cesarean section	The findings suggest no statistically significant difference in the risk of SSI between pre-incision and post-incision prophylaxis, nor between short- and long-duration prophylaxis	Critically low
Chesdachai (2022) [47]	SR and MA	Heart	PAP for more than 24 h following cardiovascular implantable electronic devices (CIED) implantation showed no significant benefit	Low
Chua (2019) [48]	SR and MA	Penis	The limited evidence available indicates that PAP has minimal effectiveness in preventing infections following hypospadia repair	Low
de Jonge (2020) [9]	SR and MA	Any surgery	There is no conclusive evidence that continuing antibiotic prophylaxis postoperatively offers any advantage over discontinuation. Following best practice standards, continuing antibiotics did not reduce the incidence of surgical site infections	Low
Klifto (2023) [49]	SR	Plastic and reconstructive	Evidence indicates that antibiotic prophylaxis is effective in preventing SSIs for certain indications and prescribed durations. PAP use has not been shown to reduce SSIs, and improper use may enhance the bacterial diversity of infections	Critically low
Murtha-Lemekhova (2022) [29]	SR and MA	Liver	PAP cannot be recommended after hepatectomy as it does not lower the risk of postoperative infections or liver-specific complications and may contribute to bacterial resistance	Low
Oppelaar (2019) [50]	SR and MA	Ear, nose, throat, and oral and maxillofacial	No significant difference was observed in postoperative infection rates between short-course (24 h and shorter) and extended-course antibiotic prophylaxis (72 h or longer) following ear, nose, throat, oral, and maxillofacial surgeries. Therefore, it is recommended to use a short course of antibiotic prophylaxis unless specific documented conditions necessitate an extended course	Critically low
Orenday-Barraza (2022) [51]	SR and MA	Spine	A meta-analysis and comprehensive literature review indicate that the routine use of postoperative antibiotics (more than 24 h) in spine surgery may not effectively prevent surgical site infections	Critically low
Phillips (2020) [30]	SR and MA	Spine	Preoperative antibiotic prophylaxis seems to offer the same level of protection against SSIs as extended PAP	Critically low
Ryan (2019) [52]	SR and MA	Any orthopedic	There is no difference in infection risk between a single dose of preoperative antibiotics and multiple doses of perioperative antibiotics for orthopedic procedures with implants	Critically low
Siddiqi (2019) [53]	SR and MA	Joint	The available evidence does not demonstrate any additional benefit from continuing PAP or extending it beyond 24 h	Critically low
Xia (2024) [31]	MA	Spine	There is no significant difference in SSI rates between patients receiving 24 h and those receiving ≥ 24 h of postoperative antibiotics. Similarly, patients with thoracolumbar drains show comparable outcomes with shorter antibiotic durations, suggesting potential cost savings and reduced hospital stays	Critically low

Key:

^a^Methodological quality was assessed using AMSTAR-2 (Assessment of Multiple Systematic Reviews) tool [40]

**Table 2:** Leveraging strategies for prevention of surgical site infection and optimizing antibiotic use in surgery

Study name	Study focus	Leveraging strategies	^a^Methodological quality
Abdallah (2024) [55]	Diabetic patients undergoing surgery	Integrating strategies such as glycemic control, antibiotic prophylaxis, and preoperative skin antisepsis. Personalized care pathways tailored to individual patient characteristics	Critically low
Ahuja (2022) [56]	Using feedback to reduce infections and optimize antibiotics in surgery	Implement robust strategies to prevent SSIs and optimize antibiotic use in surgery, including protocol refinement, real-time support for surgical teams, tailored strategies, stakeholder collaboration, ongoing education, resource provision for clinicians, and patient engagement	Low
Campos-Varela (2022) [57]	Optimal antimicrobial prophylaxis to prevent postoperative infectious complications after liver transplantation	Universal antibiotic prophylaxis is advised to prevent postoperative bacterial infections. It is recommended to tailor the choice of antibiotics to individual patients, ensuring therapy does not extend beyond 24 h	Low
Chang (2019) [58]	Antibiotic prophylaxis in the management of open fractures	Current practice and recommendations endorse early systemic antibiotic prophylaxis for open fractures of the extremities. However, variations in regimens, doses, and durations persist. Achieving consensus on optimal practices may necessitate well-designed randomized controlled trials	Critically low
Chiesa-Estomba (2019) [59]	International guidelines for perioperative antibiotic prophylaxis in head and neck surgery	In clean head and neck surgery, antibiotic prophylaxis is not routinely recommended except for neck dissection or surgeries entering the aerodigestive tract. Antibiotics should be chosen based on local resistance profiles, avoiding clindamycin in true *β*-lactam allergies In clean-contaminated procedures involving the aerodigestive tract or major reconstructions, antibiotic prophylaxis is advised for at least 24 h. For major head and neck reconstructions, including microvascular-free flaps, prophylaxis with gram-negative coverage for 24 h is recommended	Critically low
Cooper (2020) [17]	Antibiotic prophylaxis to prevent SSIs in low- and middle-income countries (LMICs)	Education to improve antibiotic prophylaxis is associated with reduction of SSIs in LMICs. However, interventions must consider local contexts and beliefs. Forming local multidisciplinary teams will encourage ownership and sustain changes	Low
Dropkin (2021) [60]	Antibiotics and inflatable penile prosthesis insertion	The benefits of a single preoperative antibiotic dose and the use of antibiotic-coated IPPs are well documented. Postoperative evidence does not support the benefit of additional antibiotics, highlighting increased risks of adverse events and drug resistance. For average-risk patients undergoing inflatable penile prosthesis (IPP) insertion, current data suggest that withholding postoperative antibiotics is safe	Critically low
Freeman (2020) [61]	Antibiotic prophylaxis for tube thoracostomy placement in trauma	For adult trauma patients needing TT insertion, we conditionally recommend administering antibiotic prophylaxis during the procedure to reduce the incidence of empyema	Low
Hussein (2022) [62]	Evaluating antibiotic prophylaxis guidelines to prevent infective endocarditis after dental procedures	Three out of four clinical practice guidelines (European Society of Cardiology, American Heart Association, National Institute of Health and Care Excellence, and Japanese Circulation Society) support that preventing infective endocarditis outweighs the risks of antibiotic resistance. Readers are advised to critically evaluate the methodology and content of these guidelines before applying them in clinical practice	Low
Jin (2021) [63]	The impact of quality improvement interventions in improving surgical infections and mortality in LMICs	SSIs in LMICs can be reduced by implementing the WHO Surgical Safety Checklist (WHO SCC). Multimodal infection control bundles and hand hygiene interventions focused on compliance can decrease hospital-acquired infections. Antimicrobial stewardship interventions also improve SSIs. Future studies should use this evidence to develop guidelines specific to LMICs	Low
Kalaria (2019) [64]	Current recommendations for antibiotic prophylaxis in plastic surgery	Systemic antibiotic prophylaxis is recommended for breast surgery, abdominoplasty, contaminated hand or face surgery, prosthetic surgery, rhinoplasty, microsurgery, and burn reconstruction. Despite this, many plastic surgeons still use prophylactic antibiotics in clean cases, often following their own protocols. Clear guidelines for antibiotic use in plastic surgery await results from randomized controlled trials	Critically low
Valeska Martinez‑Sobalvarro (2022) [65]	Antimicrobial stewardship for surgical antibiotic prophylaxis and SSIs	Antimicrobial stewardship strategies, including audit, feedback, education, protocol implementation, and computer-assisted decision support, show effectiveness in promoting adherence to surgical antibiotic prophylaxis protocols and reducing surgical site infection rates, with positive economic implications. However, additional randomized clinical trials are needed to strengthen the evidence base and support decision-making	Low
Righi (2023) [66]	Guidelines on perioperative antibiotic prophylaxis for patients colonized by multidrug-resistant gram-negative bacteria	Reducing SSIs requires integrating effective antibiotic interventions and best surgical practices, including minimizing operative time, regulating glucose and temperature, optimizing sterile techniques, and managing patient comorbidities. Enhancing antibiotic stewardship is essential to monitor prescription patterns, improve surveillance, ensure guideline adherence, and promote a multidisciplinary approach to tackle SSIs	Low
Woodfield (2022) [26]	Strategies for antibiotic administration for bowel preparation among patients undergoing elective colorectal surgery	This study demonstrated that adding oral antibiotics (OA) to intravenous (IV) antibiotics reduced incisional SSI by more than 50%. The findings support using OA in conjunction with IV antibiotics to decrease incisional SSIs in elective colorectal surgery patients	Low

Key:

^a^Methodological quality was assessed using AMSTAR-2 (Assessment of Multiple Systematic Reviews) tool [40]

### Assessment of publication bias and risk of bias

#### Publication bias

Publication bias in the reviews included in the meta-analysis was evaluated using a funnel plot [39]. In this plot, the treatment effect estimates from individual reviews (e.g., risk ratios) were plotted against their standard errors.

#### Risk of bias (methodological quality)

Two reviewers (G. G. N. and B. J. N.) independently assessed the methodological quality of the included reviews using the AMSTAR-2 (Assessment of Multiple Systematic Reviews) tool [40]. Any disagreements were resolved by consulting a third reviewer (G. M. B.). We evaluated 16 domains, which include 7 critical domains (questions 2, 4, 7, 9, 11, 13, and 15). Compliance with each domain was rated as “yes,” “partial yes,” or “no.” The overall quality of the reviews was categorized into four levels as high, moderate, low, and critically low based on compliance with critical and noncritical domains.

### Narrative synthesis

For the narrative synthesis process, two independent reviewers (G. M. B. and G. G. N.) conducted a thorough assessment of the included reviews to identify key themes and summarize findings. The reviewers independently extracted data and grouped it into thematic areas that were relevant to the study objectives. If there were discrepancies in data coding or thematic classification, a third reviewer was consulted to resolve conflicts and ensure consistency in the synthesis (M. V. M.). The narrative synthesis was undertaken to explore strategies for preventing SSIs and optimizing antibiotic use in surgical settings. No a priori themes were established; instead, themes were developed iteratively based on the relevance of study findings to the research questions. This synthesis involved systematically extracting and analyzing qualitative and quantitative data from the included reviews.

### Statistical analysis

Risk ratio (RR) was used to estimate the effect of continuing antibiotic prophylaxis versus its discontinuation on the occurrence of SSIs, with a fixed-effects model (Mantel–Haenszel method) applied to pool the data and a 95% confidence interval (95% CI). Risk ratios and 95% CIs were calculated based on the total number of events and patients across all studies in each review. *I*^2^ was used to estimate the heterogeneity of the pooled reviews*. I*^2^ value greater than 50% indicated high heterogeneity in the estimates [41]. We used the fixed-effects model when there was low to moderate heterogeneity between reviews [42]. In this review, we defined the discontinuation of post-exposure prophylaxis (PAP) as the cessation of antibiotic prophylaxis immediately following the closure of the surgical incision or the use of antibiotics for no more than 24-h post-surgery [43]. We performed a subgroup analysis based on the surgical sites [44]. All quantitative statistics were performed using R software version 4.2.3. Significant results were considered when the 95% CI did not include one, or the *p*-value was less than 0.05.

## Results

### Study selection

In our umbrella review, we initially screened 1156 articles. After applying eligibility criteria, we identified 28 review articles suitable for final analysis. These articles encompassed over 457 primary studies, involving a total of 80,483 patients from 9 meta-analysis reviews, which were used for estimating the pooled effect measure (RR) (Fig. 1).

### Study characteristics

Overall, 28 review articles qualified for final analysis [9, 17, 26, 29–32, 45–65]. Out of 28 reviews, 14 focused on comparing the effectiveness of discontinued PAP versus its continuation in preventing SSIs (Table 1), while 13 articles synthesized evidence to improve best practices (strategies), particularly PAP in surgical settings (Table 2). One review by Cooper et al. (2020) [17] documented both the effectiveness of PAP and the strategies to reduce SSIs in surgical settings. As shown in Table 1, discontinuation and continuation of PAP, especially beyond 24 h, demonstrated comparable outcomes in reducing the incidence of SSIs [9, 17, 29–32, 45–53], irrespective of the surgical site or procedure. None of the reviews had a high or moderate quality. The majority of reviews (16) were rated as having critically low quality, and 12 reviews were classified as low quality. The poor methodological quality was mainly due to the absence and justification of a list of potentially relevant studies that were excluded after full-text screening which is a critical domain (item number 7). Additionally, half of the reviews (14) did not register their protocol before conducting a review, and only one review addressed the funding sources for the studies included in the review (Supplementary file 2).

### Meta-analysis

**Fig. 2:**
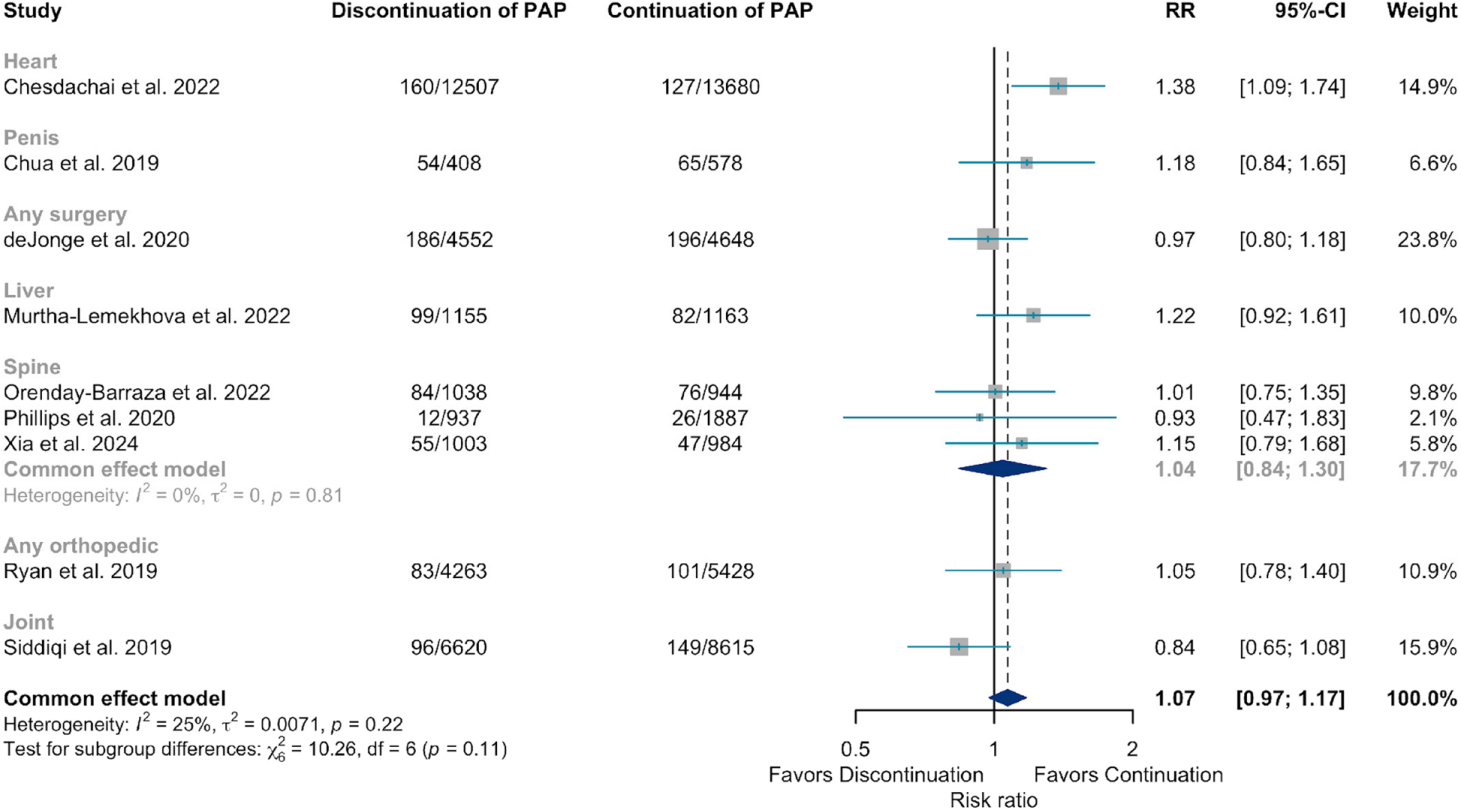
Meta-analysis to estimate the risk ratio (RR) between discontinuation of postoperative antibiotic prophylaxis (PAP) within 24-h post-surgery and continuation of PAP for preventing surgical site infections (SSI)

Nine meta-analyses compared the effectiveness of preventing SSIs between discontinuation of PAP versus its continuation beyond 24-h post-surgery. Since de Jonge (2020) [9] reported both best practices and non-best practices, meta-analysis was conducted for reviews that adhered to the best practices, while sensitivity analysis was performed by including studies that did not follow the best practices. The results showed a risk ratio (RR) of 1.07 (95% *CI*: 0.97–1.17, *I*^2^: 25%, *p*-value: 0.22). The RR of 1.07 shows that the intervention group (discontinuation of PAP within 24-h post-surgery reduces) has a 7% higher risk of having SSIs compared to the control group (continuation of PAP). However, the 95% CI includes 1, indicating that the risk reduction is not statistically significant. The value of *I*^2^: 25% indicates low to moderate variability among reviews (Fig. 2). In this review, one review by Chesdachai (2022) [47] involving heart surgeries favored the continuation of PAP (*RR*: 1.38, 95% *CI*: 1.09–1.74). Moreover, when the review by de Jonge (2020) [9] was included, even with studies not adhering to best practices, the outcome favored the continuation of PAP (*RR*: 1.12, 95% *CI*: 1.03–1.21, *p*-value: 0.30) (Supplementary file 3).

### Funnel plot

**Fig. 3:**
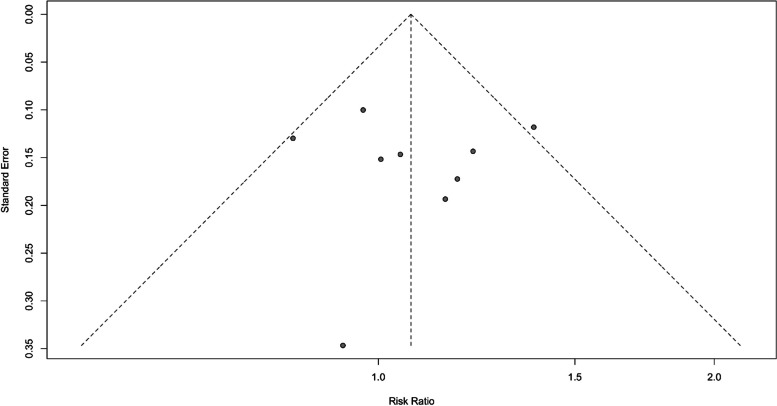
Funnel plot showing the publication bias for studies included in the meta-analysis

Ideally, the plot resembled a symmetrical inverted funnel, with larger reviews with smaller standard errors clustering around the average treatment effect at the top and smaller reviews with larger standard errors scattering more widely at the bottom. In contrast, asymmetry may suggest potential bias, such as the selective publication of reviews where smaller or less favorable reviews might have been underrepresented (Fig. 3).

### Leveraging strategies to prevent SSI and optimize antibiotic use in surgical settings

#### Reassessing and refining guidelines to meet the specific needs

This synthesis underscored a pressing requirement to update guidelines in accordance with current evidence and evolving clinical practices. Key findings emphasized considerable variability in guideline effectiveness across diverse healthcare settings, with persistent variations observed in regimens, doses, and durations [57, 61], highlighting the important role of customized implementation strategies in bolstering adherence and overall efficacy [62]. Moreover, the review identified notable evidence gaps, indicating that randomized clinical trials (RCTs) are still needed to strengthen the evidence base and support decision-making [57, 63, 64], including studies that will help explore effective strategies for enabling de-implementation of PAP practice, particularly considering specific contextual and patient factors [17, 54, 56, 58, 61, 62].

### Multidisciplinary collaboration

Providing real-time multidisciplinary support to surgical teams can help to prevent SSIs [17, 55, 62, 64]. This approach ensures that specialists from various fields, such as infectious disease experts, pharmacists, and infection control nurses, are readily available to offer their expertise. Multidisciplinary collaboration allows for comprehensive assessments of infection risks tailored to individual patients and surgical contexts. Moreover, it facilitates immediate consultation on antibiotic stewardship and optimizing infection prevention efforts during surgeries.

### Availability of resources needed for best practices

The availability of resources necessary for best practices in infection prevention is critical for mitigating SSIs [55, 64, 65]. These resources include surgical rooms, sterile surgical equipment, effective antimicrobial agents, and comprehensive infection control protocols. Having these resources readily accessible ensures that surgical teams can adhere to stringent hygiene standards, optimize surgical techniques, and minimize contamination risks during procedures. Furthermore, access to up-to-date guidelines and educational materials supports ongoing training and awareness among healthcare providers.

### Educating on SSI prevention and antibiotic stewardship

Education on SSI prevention and antibiotic stewardship ensures that surgical teams are equipped with the latest evidence-based guidelines and strategies to prevent SSIs [17, 55, 64]. This includes proper antimicrobial use, adherence to sterile techniques, and awareness of risk factors contributing to infections. Moreover, education fosters a culture of continuous improvement, encouraging ongoing learning and adaptation to emerging best practices in infection prevention. It empowers healthcare professionals to implement proactive measures and respond effectively to challenges during surgical procedures.

### Patient education

Engaging and educating patients in their care empower them to understand and adhere to infection prevention measures [17, 55, 65]. This includes educating patients about the importance of hand hygiene, wound care, and the proper use of antibiotics. Informed patients can actively participate in shared decision-making regarding their treatment plans, ensuring better compliance with postoperative instructions that minimize infection risks. Furthermore, patient involvement promotes transparency and trust between healthcare providers and patients. By fostering open communication, healthcare teams can address patient concerns, manage expectations, and collaborate effectively to prevent SSIs.

## Discussion

To the best of the authors’ knowledge, this is the first umbrella review to synthesize evidence on PAP practice following the WHO recommendations of 2018 [12]. In our umbrella review, we found that prolonging antibiotic prophylaxis beyond 24-h post-surgery did not demonstrate any additional benefits in reducing the occurrence of SSIs (*RR*: 1.07, 95% *CI*: 0.97–1.17). The analysis encompassed 80,483 patients from 9 meta-analyses. These results indicate that the critical period for effective antibiotic prophylaxis is during the preoperative and intraoperative periods, beyond which continued use does not significantly impact SSI rates. Our findings are consistent with existing guidelines from major health organizations such as the WHO and the CDC. Both organizations recommend limiting antibiotic prophylaxis to the perioperative period [12, 13], typically immediately after surgical incision closure for clean and clean-contaminated surgeries, regardless of the type of procedure [12].

The pooled estimate (*RR*: 1.07) and the upper limit of the confidence interval (95% *CI*: 0.97–1.17) suggest that while there is no statistical significance at the population level, the evidence still favors the benefit of PAP practice at an individual level (personalized approach), supporting the proposal that extending PAP beyond 24-h post-surgery should be guided by other evidence such as patient-specific factors [22]. On the other hand, not extending PAP offers several key benefits, primarily by reducing the risk of developing antibiotic-resistant bacteria [66], a major public health concern [67, 68]. Moreover, limiting antibiotic use to the preoperative and intraoperative periods also decreases the likelihood of adverse drug reactions, enhancing patient safety [69]. Additionally, this approach results in significant cost savings for the patients and healthcare systems by reducing the expenses associated with unnecessary antibiotic use and its complications [70]. More importantly, this practice aligns with evidence-based guidelines from major health organizations, ensuring adherence to the best practices in surgical care [12].

Despite the existing evidence supporting the discontinuation of PAP in surgical settings [12], the practices that are not guided by evidence still persist, particularly in LMICs [14–16, 71]. There remains a critical need to implement effective interventions aimed at improving SSI prevention and optimizing antibiotic use in these settings. Addressing these challenges requires tailored interventions that consider the unique socioeconomic and healthcare landscapes of LMICs [18]. This includes prioritizing capacity-building initiatives, enhancing healthcare infrastructure, and promoting sustainable practices that align with local contexts. In addressing these challenges, this review recommends several strategies to guide the design and implementation of effective interventions. Firstly, regularly assessing and refining guidelines tailored to specific surgical contexts and patient characteristics. This ensures that guidelines remain responsive to changing clinical needs and demographic factors, thereby enhancing their effectiveness in reducing SSIs [72]. Secondly, fostering multidisciplinary collaboration. By bringing together surgical teams, infectious disease specialists, microbiologists, pharmacists, and nurses, comprehensive approaches to SSI prevention and antibiotic stewardship can be co-developed and implemented [73]. This collaborative effort promotes knowledge sharing, improves adherence to guidelines, and facilitates the implementation of evidence-based practices that ultimately enhance patient outcomes and reduce the incidence of SSIs in surgical settings.

Effective implementation of guidelines for PAP discontinuation relies heavily on the availability of adequate resources, including staffing, technological support, and antimicrobial stewardship programs [74]. Ensuring that surgical teams have access to the necessary resources can facilitate adherence to updated guidelines and enhance overall patient care. Additionally, continuous education and training programs should be implemented to keep surgical teams and other healthcare providers abreast of current evidence and guidelines [18]. Educating patients about the rationale for discontinuing antibiotics and involving them in the decision-making process can improve adherence to postoperative care plans and reduce anxiety or misconceptions about antibiotic use [10]. These strategies collectively support a holistic approach to improving care quality and patient safety across healthcare environments.

One limitation of the current review is the absence of an assessment comparing the impact of adherence to best practices on preventing SSIs. During our meta-analysis, we identified only one review by de Jonge et al. (2020) [9] that conducted a subgroup analysis comparing adherence versus nonadherence to best practices about discontinued or continued use of PAP to prevent SSIs. In this study, being adherent (*RR*: 1.04, 95% *CI*: 0.85–1.27) showed no significant difference in benefits, while not being adherent (*RR*: 0.79, 95% *CI*: 0.67–0.94) to best practices favored the continuation of PAP. Furthermore, sensitivity analysis, including studies that did not adhere to the best practices reported by de Jonge et al. (2020) [9], showed that the overall benefits favored the continuation of PAP practice (*RR*: 1.12, 95% *CI*: 1.03–1.21, *p*-value: 0.30). Another limitation is that we did not exclude the possibility of one article contributing to multiple reviews [44]. Given the nature of umbrella reviews, a single original article may be included in different reviews [34].

## Conclusions

Our study found no significant benefits to patients from continuing PAP beyond 24 h compared to immediate discontinuation. This suggests that current practices of extending PAP may not provide additional protective effects against SSIs. Our study supports the WHO recommendations of 2018 advocating for the immediate discontinuation of PAP following surgical incision closure in clean and clean-contaminated surgeries. Future research should focus on longitudinal assessments to compare long-term outcomes, including antibiotic resistance patterns, between immediate discontinuation and prolonged PAP across various surgical settings. Cost-effectiveness analyses are needed to evaluate economic impacts, while patient-centered studies should explore satisfaction and adherence to antibiotic stewardship. Additionally, more studies such as de-implementation research should be conducted to refine guidelines tailored to specific needs such as surgical contexts and patient characteristics to optimize SSI prevention and antibiotic use.

## Supplementary information

Supplementary Material 1. Search queries for PubMed and Scopus.

Supplementary Material 2. Methodological quality assessment.

Supplementary Material 3. Sensitivity analysis. Meta-analysis to estimate the risk ratio (RR) between discontinuation of postoperative antibiotic prophylaxis (PAP) within 24 h post-surgery and continuation of PAP for preventing surgical site infections (SSI) after including all studies (those adhered and not adhered to the best practice) by de Jonge (2020) [9].

## Data Availability

Data used to draw this conclusion are available from the corresponding author on reasonable request.

## Abbreviations

LMICs: Lower- and middle-income countries
PAP: Postoperative antibiotic prophylaxis
RR: Risk ratio
SSI: Surgical site infection
WHO: World Health Organization
