# Supplementary material for: Synthesizing evidence to guide the design and implementation of effective strategies for discontinuing postoperative antibiotic prophylaxis in surgical settings: an umbrella review post-WHO 2018 recommendations
Source: Syst Rev. 2025 Jan 8;14:7. doi: 10.1186/s13643-024-02750-7 (PMC11708070; doi:10.1186/s13643-024-02750-7)
Supplement: Supplementary file 1 — Supplementary Material 1. Search queries for PubMed and Scopus. [file 13643_2024_2750_MOESM1_ESM.docx]

**Synthesizing evidence to guide the design and implementation of effective strategies for discontinuing postoperative antibiotic prophylaxis in surgical settings: an umbrella review post-WHO 2018 recommendations**

George Msema Bwire, Renatus B. Magati, Hafidhi H. Ntissi, Tusaligwe Mbilinyi, Martine A. Manguzu, Goodluck G. Nyondo, Belinda J. Njiro, Lilian B. Nkinda, ﻿Castory G. Munishi, Obadia Nyongole, Pacifique Ndayishimiye^,^,Mtebe V. Majigo

**Supplementary file 1**: Search queries for PubMed and Scopus

| **Database** | **Search query** |
| --- | --- |
| PubMed/MEDLINE | ("Postoperative antibiotic prophylaxis" OR "Antibiotic prophylaxis" OR "Surgical infections") [MeSH Terms] OR ("Postoperative antibiotic prophylaxis" OR "Antibiotic prophylaxis" OR "Surgical infections") [All Fields] AND ("Systematic review" OR "Meta-analysis" OR "Evidence synthesis" OR "Literature review") [Publication Type] AND ("Best practices" OR "Guidelines" OR "Recommendations" OR "Strategies" OR "Interventions") [Title/Abstract])  Date when a search was conducted: 05^th^ July 2024 |
| Scopus | (TITLE-ABS("Postoperative antibiotic prophylaxis" OR "Antibiotic prophylaxis" OR "Surgical infections") AND TITLE-ABS("Systematic review" OR "Meta-analysis" OR "Evidence synthesis" OR "Literature review") AND TITLE-ABS("Best practices" OR "Guidelines" OR "Recommendations" OR "Strategies" OR "Interventions"))  Date when a search was conducted: 05^th^ July 2024. |
