# Supplementary material for: Synthesizing evidence to guide the design and implementation of effective strategies for discontinuing postoperative antibiotic prophylaxis in surgical settings: an umbrella review post-WHO 2018 recommendations
Source: Syst Rev. 2025 Jan 8;14:7. doi: 10.1186/s13643-024-02750-7 (PMC11708070; doi:10.1186/s13643-024-02750-7)
Supplement: Supplementary file 3 — Supplementary Material 3. Sensitivity analysis. Meta-analysis to estimate the risk ratio (RR) between discontinuation of postoperative antibiotic prophylaxis (PAP) within 24 h post-surgery and continuation of PAP for preventing surgical site infections (SSI) after including all studies (those adhered and not adhered to the best practice) by de Jonge (2020) [9]. [file 13643_2024_2750_MOESM3_ESM.docx]

**Synthesizing evidence to guide the design and implementation of effective strategies for discontinuing postoperative antibiotic prophylaxis in surgical settings: an umbrella review post-WHO 2018 recommendations**

George Msema Bwire, Renatus B. Magati, Hafidhi H. Ntissi, Tusaligwe Mbilinyi, Martine A. Manguzu, Goodluck G. Nyondo, Belinda J. Njiro, Lilian B. Nkinda, ﻿Castory G. Munishi, Obadia Nyongole, Pacifique Ndayishimiye^,^,Mtebe V. Majigo

**Supplementary file 3.** Sensitivity analysis. Meta-analysis to estimate the risk ratio (RR) between discontinuation of postoperative antibiotic prophylaxis (PAP) within 24 hours post-surgery and continuation of PAP for preventing surgical site infections (SSI) after including all studies (those adhered and not adhered to the best practice) by de Jonge (2020) [9].

**
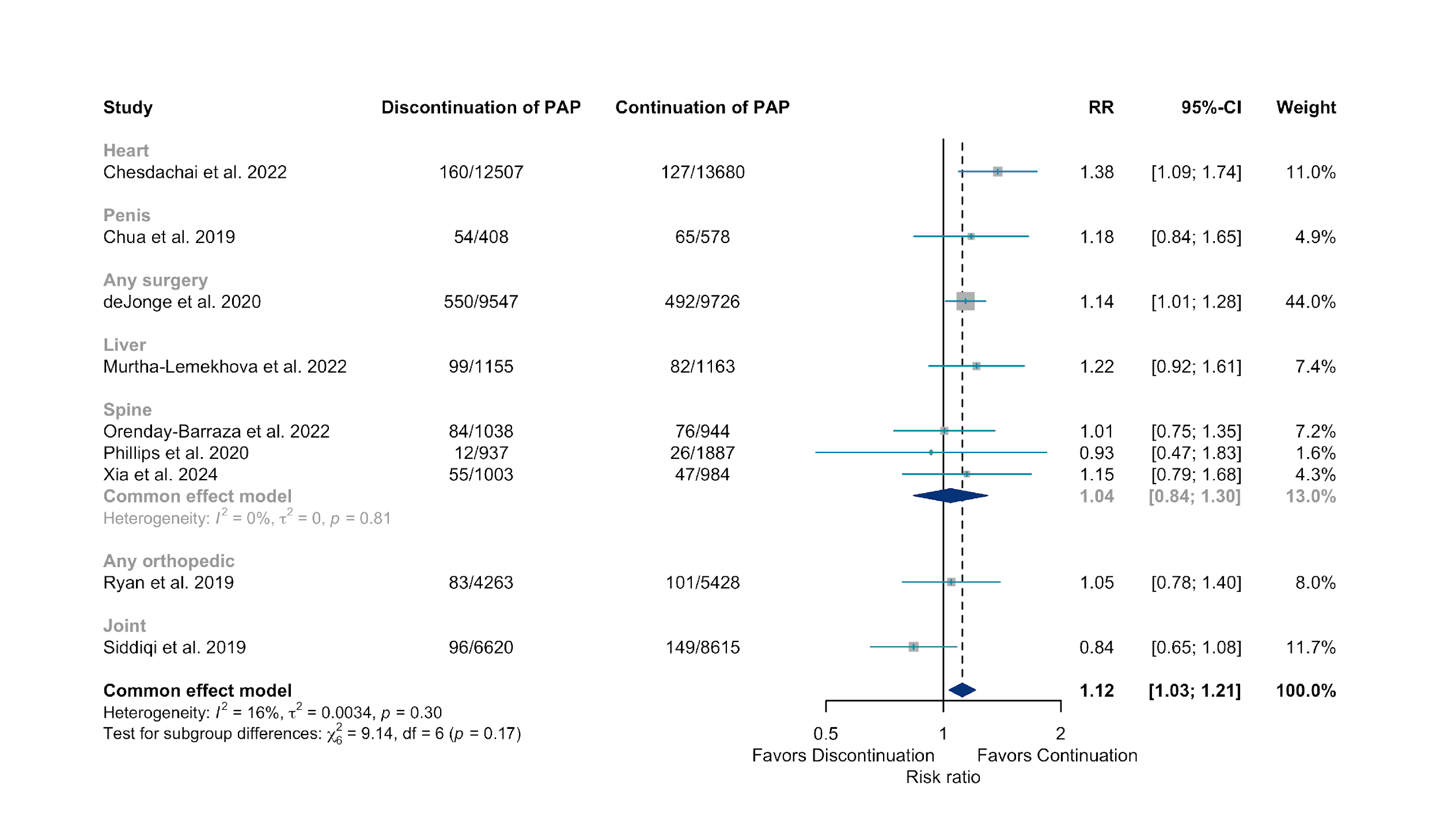
**
